# Drosophila eugracilis – Akt

**DOI:** 10.17912/micropub.biology.000544

**Published:** 2022-07-02

**Authors:** Ashley Morgan, Cole A. Kiser, Isabel Bronson, Hanjun Lin, Nicholas Guillette, Robert McMahon, Jennifer A. Kennell, Lindsey J Long, Laura K. Reed, Chinmay P. Rele

**Affiliations:** 1 The University of Alabama, Tuscaloosa, AL USA; 2 Vassar College, Poughkeepsie, NY USA; 3 Oklahoma Christian University, Edmond, OK USA

## Abstract

Gene Model for *Akt* in the *D. eugracilis* (DeugGB2) assembly (GCA_000236325.2).

**Figure 1.**
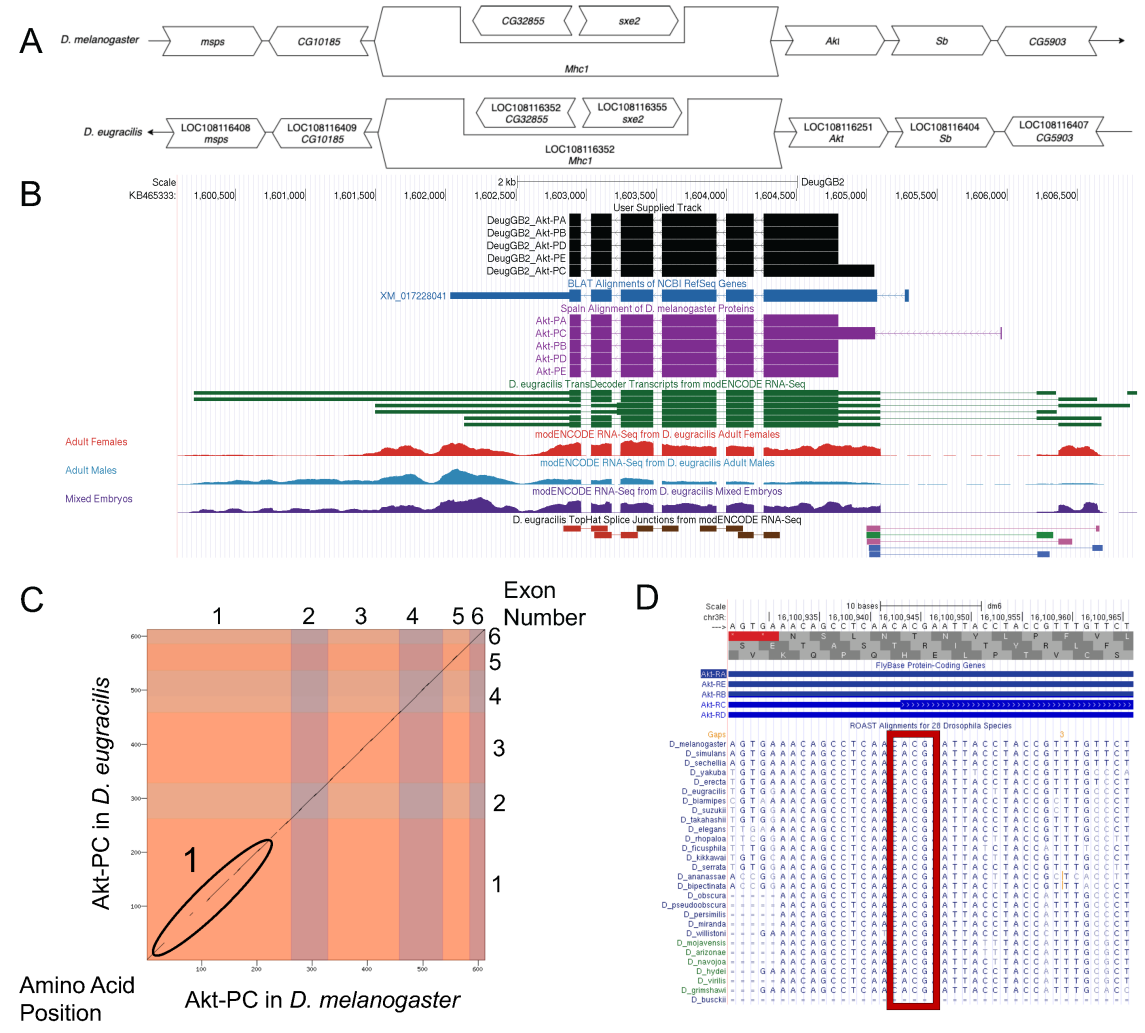
(A) Synteny of genomic neighborhood of *Akt* in both *D. melanogaster* as well as *D. eugracilis* . Gene arrows pointing in the same direction as *Akt* in both *D. eugracilis* and *D. melanogaster* are on the same strand as *Akt* ; gene arrows pointing in the opposite direction are on the opposite strand. The thin underlying arrow in *D. melanogaster* that points to the right indicates *Akt* is on the + strand and the thin underlying arrow pointing to the left in *D. eugracilis* indicates *Akt* is on the – strand. White arrows in *D. eugracilis* indicate orthology to the corresponding gene in *D. melanogaster* . *CG32855* and *sxe2* are nested within an intron of *Mhc1* in both species. The gene names given in the *D. eugracilis* gene arrows indicate the orthologous gene in *D. melanogaster* , while the locus identifiers are specific to *D. eugracilis* ; (B) Gene Model in UCSC Track Hub (Raney *et al.,* 2014): The gene model in *D. eugracilis* (black), Spaln of D. melanogaster Proteins (purple, alignment of refseq proteins from *D. melanogaster* ), BLAT alignments of NCBI RefSeq Genes (blue, alignment of refseq genes for *D. eugracilis* ), RNA-Seq from adult females (red), adult males (blue), and mixed embryos (purple) (alignment of Illumina RNAseq reads from *D. eugracilis* ), and Transcripts (green) including coding regions predicted by TransDecoder and Splice Junctions Predicted by regtools using *D. eugracilis* RNA-Seq (Chen *et al.,* 2014; PRJNA63469). Splice junctions shown have a minimum read-depth of 10 with 10-49, 50-99, 100-499, 500-999, >1000 supporting reads in blue, green, pink, brown, and red respectively. The custom gene model (User Supplied Track) is indicated in black with exons depicted with boxes and introns with narrow lines (arrows indicate direction of transcription). (C) Dot Plot of amino acid identity for Akt-PC in *D. melanogaster* ( *x* -axis) vs. Akt-PC in *D. eugracilis* ( *y* -axis). Amino acid number is indicated along the left and bottom; exon number is indicated along the top and right. Each colored rectangle represents an exon. Region 1 indicates a lack of sequence similarity between the two sequences; (D) ROAST Alignments Conservation track within the UCSC Genome Browser. The ROAST Alignments for 28 Drosophila Species track within the UCSC Genome Browser shows the genomic region surrounding the beginning of the Akt-PC isoform in all 28 species shown, demonstrating that the non-canonical start codon for Akt-PC is conserved across the genus.

## Description

Introduction

The insulin signaling pathway is a highly conserved pathway in animals, and is central to nutrient uptake (Hietakangas and Cohen 2009, Grewal 2009). Akt kinase ( *Akt* also known as *Akt1, Protein Kinase B, PKB;* FBgn0010379) regulates stress response, aging, and cell growth and survival in *Drosophila* (Stavely *et al* ., 1998; Verdu *et al.* , 1999). It is involved in signal transduction pathways in physiological and neurological pathways in *Drosophila* (Guo and Zhong 2006). It encodes a core serine-threonine kinase (Bellacosa et al. 1991) component of the Insulin-like growth factor pathway that functions downstream of, and following its activation by the *Pi3K92E* product in *Drosophila* (Andjelkovic et al., 1995). It is activated by phosphatidylinositol binding and phosphorylation (Potter *et al* ., 2002). The gene model reported ( *Deug_Akt* ) was determined in the Apr. 2013 (BCM-HGSC/Deug_2.0; GCA_000236325.2) of *D. eugracilis* and compared to the ortholog *dmel_Akt* (GCA_000001215.4, FB2021_02; Larkin *et al.,* 2021). *D. eugracilis* is part of the *melanogaste* r species group within the subgenus *Sophophora* of *Drosophila* (Pélandakis et al., 1993). It was first described as *Tanygastrella gracilis* by Duda (1924) and revised to *Drosophila eugracilis* by Bock and Wheeler (1972). *D. eugracilis* is found in humid tropical and subtropical forests across southeast Asia (https://www.taxodros.uzh.ch). The methods and dataset versions used to establish the gene model are described in Rele *et al* . (2020). The Genomics Education Partnership maintains a mirror of the UCSC Genome Browser (Kent WJ *et al* ., 2002; Gonzalez *et al* ., 2021), which is available at http://gander.wustl.edu .The predicted gene model in *D. eugracilis* for *Akt* was found in NCBI RefSeq Accession XM_017228041.1 and Locus ID LOC108116251.

Synteny

*Akt* is located on chromosome 3R (Muller element D) in *D. melanogaster* and is surrounded by *sxe2, CG32855,* and *Mhcl* (upstream) and *Sb* and *CG5903* (downstream). After performing a *tblastn* , the putative *Akt* ortholog (LOC10811625/XM_017228041.1/XP_017083530.1, e-value of 0.0 and percent identity of 90.39%) in *D. eugracilis* was found to be on scaffold KB465333 (mapped to Muller element D) and is surrounded by orthologs to *sxe2* (LOC108116355/XM_017228178.2/XP_017083667.2, e-value of 0.0 and percent identity of 87.08%), *Mhcl* (LOC108116352/XM_017228167.2/XP_017083656.2, e-value of 0.0 and percent identity of 97.13%), *Sb* (LOC108116404/XM_017228281.2/XP_017083770.2, e-value of 0.0 and percent identity of 87.02%), and *CG5903* ( *Mic26-27* /LOC108116407/XM_017228287.1/XP_017083776.1, e-value of 7e-151 and percent identity of 88.26%) as determined by *blastp* (Figure 1A, Altschul *et al.* , 1990). The annotated model is likely to be the true ortholog of *Akt* due to the high level of synteny that exists between *D. melanogaster* and *D. eugracilis* , and the reciprocal best *blast* hits between the two genes.

Protein Model

There are five RNA isoforms of *Akt* : *Akt-RA, Akt-RB, Akt-RC, Akt-RD,* and *Akt-RE* . The RA, RB, RD, and RE isoforms have identical protein coding sequences, represented by the Akt-PE protein isoform here. The Akt-PC coding sequence is unique. Both the Akt-PC and Akt-PE isoforms in *D. melanogaster* are encoded by six coding exons. The Akt-PC and Akt-PE isoforms in *D. eugracilis* are encoded by six coding exons **(** Figure 1B). The coordinates of the curated gene models can be found in NCBI at GenBank/BankIt using the accessions BK059589, BK059590, BK059591, BK059592, and BK059593, one for each protein-coding isoform of Akt **.** These data are also available in Extended Data files below, which are archived in CaltechData.

Special characteristics of the gene model

**Non-canonical Start Codon:** The *Akt-RC* isoform has a non-canonical ACG start codon. This is well conserved across the 28 *Drosophila* species as shown in Figure 1D. This non-canonical start codon is used for the translation of the Akt-PC isoform in *D. melanogaster* , (Figure 1D). There is a high level of conservation of the non-canonical ACG start codon (encoding threonine) across all 28 *Drosophila* species, as well as high conservation of the region surrounding the non-canonical start codon. This provides evidence for the existence of a non-canonical start codon in the Akt-PC isoform in *D. eugracilis* .

## Methods

Detailed methods including algorithms, database versions, and citations for the complete annotation process can be found in Rele *et al.* (2020).

## Reagents

NA

## Extended Data

Description: GTF. Resource Type: Model. DOI: 10.22002/D1.20201

Description: FAA. Resource Type: Model. DOI: 10.22002/D1.20202

Description: FNA. Resource Type: Model. DOI: 10.22002/D1.20203
